# The effect of the MyBFF@school program on cardiorespiratory fitness in overweight and obese primary schoolchildren: a cluster randomized controlled trial

**DOI:** 10.1186/s12889-024-20723-2

**Published:** 2025-01-09

**Authors:** Abdul Halim Mokhtar, Muhammad Ashaari Kamarudin, Alston Choong, Lakvinder Singh, Vinotha Genisan, Abqariyah Yahya, Ruziana Mona Wan Mohd Zin, Fuziah Md. Zain, Rusidah Selamat, Zahari Ishak, Muhammad Yazid Jalaludin

**Affiliations:** 1 Department of Sports Medicine, Faculty of Medicine, Universiti Malaya, Kuala Lumpur, Wilayah Persekutuan Kuala Lumpur 50603 Malaysia; 2 Faculty of Sports and Exercise Science, Universiti Malaya, Kuala Lumpur, Wilayah Persekutuan Kuala Lumpur 50603 Malaysia; 3 University of Malaya Consultancy Unit, Universiti Malaya, Kuala Lumpur, Wilayah Persekutuan 50603 Malaysia; 4 Department of Social and Preventive Medicine, Faculty of Medicine, Universiti Malaya, Kuala Lumpur, Wilayah Persekutuan 50603 Malaysia; 5 Department of Pediatrics, Faculty of Medicine, Universiti Malaya, Kuala Lumpur, Wilayah Persekutuan 50603 Malaysia; 6 Endocrine and Metabolic Unit, Nutrition, Metabolic & Cardiovascular Research Centre, Institute for Medical Research, National Institute of Health (NIH), Ministry of Health, Setia Alam, 40170 Malaysia; 7 Department of Pediatrics, Putrajaya Hospital, Ministry of Health, Jalan P9, Pusat Pentadbiran Kerajaan Persekutuan Presint 7, Putrajaya, Wilayah Persekutuan 62250 Malaysia; 8 Nutrition Division, Level 1, Block E3, Complex E, Ministry of Health, Federal Government Administrative Centre, Putrajaya, Wilayah Persekutuan 62590 Malaysia; 9 FOSSLA, UCSI University, Kuala Lumpur, 56000 Malaysia

**Keywords:** Primary School Children, Overweight, Obesity, Intervention, Physical fitness

## Abstract

**Background:**

MyBFF@school program consisting physical activity in the formed small-sided games (SSG), nutrition and psychology education was designed to combat obesity among schoolchildren in Malaysia. It was expected to improve cardiorespiratory fitness, hence, contributing to obesity treatment and prevention. Thus, we aimed to study the effects of the MyBFF@school program on the cardiorespiratory fitness of overweight and obese primary schoolchildren.

**Methods:**

Twenty-three out of 1196 government primary schools in central Peninsular Malaysia participated in this cluster-randomized control study. Schoolchildren aged 9–11 years with a body mass index (BMI) *z*-score greater than + 1 SD (WHO) were eligible for the study. The intervention group participated in the MyBFF@school program while the control followed the existing standard curriculum. The primary outcome was cardiorespiratory fitness using physical fitness score (PFS) measured by the modified Harvard step test. Data were collected at baseline, month-3 and month-6 and were analyzed according to the intention-to-treat principle using mixed linear models.

**Results:**

A total of 954 schoolchildren completed six months follow up, with 439 (*n* = 439) in the intervention group (*n* = seven schools), while 515 (*n* = 515) in the control group (*n* = 16 schools). In the first three months, there was significant within-group PFS improvement in overall (both), girls (both) and obese (control). Comparing between-groups, the mean differences favored the control in most parameters, but were not significant: overall (-0.15(-0.75, 0.45), *p* = 0.83), boys (-0.07(-0.98, 0.83), *p* = 0.83), girls (-0.27(-1.27, 0.73), *p* = 0.81), overweight (-0.16(-1.28, 0.94), *p* = 0.97), obese (-0.05(-1.03, 0.92), *p* = 0.93), morbidly obese (-0.68(-2.43, 1.05), *p* = 0.26), urban (0.07(-0.79, 0.94), *p* = 0.45), and rural (-0.35(-1.34, 0.62), *p* = 0.30). At month-six, the within-group improvements maintained. However, the mean differences now favored the intervention group although they remained not significant: overall (0.05(-0.98, 1.07), *p* = 0.69), boys (0.06(-1.35, 1.46), *p* = 0.86), girls (0.10(-1.31, 1.51), *p* = 0.74), overweight (0.15(-1.07, 1.36), *p* = 0.93), obese (0.28(-0.98, 1.55), *p* = 0.75), morbidly obese (-0.79(-2.74, 1.15), *p* = 0.47), urban (0.61(-0.56, 1.77), *p* = 0.47), and rural (-0.69(-2.52, 1.14), *p* = 0.17).

**Conclusions:**

MyBFF@school program showed positive trend in cardiorespiratory fitness changes especially after six months. MyBFF@school intervention program has the potential to combat obesity in primary schoolchildren and should be at least six months.

**Trial registration:**

Clinical trial number: NCT04155255, November 7, 2019 (Retrospective registered). National Medical Research Register: NMRR-13-439-16563. Registered July 23, 2013. The intervention program was approved by the Medical Research and Ethics Committee (MREC), Ministry of Health, Malaysia and, the Educational Planning and Research Division (EPRD), Ministry of Education, Malaysia. It was funded by the Ministry of Health, Malaysia.

## Background

Obesity is currently a serious global issue. The World Health Organization (WHO) stated that at least 2.8 million people die each year resulting from overweight or obesity. The mortality rate increases with degrees of overweight as measured by body mass index (BMI) [1]. Furthermore, a systematic evaluation had found a rapid increase in elevated BMI and the related disease burden since the last three decades [2]. For children aged 5–19 years, overweight is defined by their BMI for age (BMI *z*-score) of greater than 1 standard deviation (SD) above the WHO Growth Reference Median, while obese has more than 2 SD above Growth Reference Median [3].

The prevalence of overweight and obese individuals is also escalating rapidly in numerous Asian countries, including Malaysia. The Malaysian National Health and Morbidity Survey (NHMS) 2011 found the prevalence rate of obesity for children aged below 18 years-old was 5.7% [4]. Subsequent NHMS survey (2015) showed that the prevalence rate has increased to 11.9% [5] and latest (NHMS, 2019) revealed the rate of 29.8% (26.2% of girls and 33.2% of boys) [6]. This worrying situation requires urgent attention and prompt action.

School-based programs have been shown to be effective in preventing and treating overweight and obesity in schoolchildren and have been recommended in several guidelines [7, 8]. Lifestyle intervention based on nutrition, behavior changes, and exercise are commonly used to treat obesity in children [8, 9]. Exercise increases physical activity which is negatively associated with the risk of obesity [10]. Unfortunately, intervention to increase physical activity generally yields weak or no effect [11]. Hence, there is a need to find an effective intervention that includes the promotion of physical activity [12]. We introduced the MyBFF@school program to combat obesity in Malaysian schoolchildren. The program was composed of physical activity, nutrition and psychology educational components. The physical activity consisted of small-sided games in which the overweight and obese children were assigned into small groups and played football, handball and fun games [13]. The SSG would promote physical activity, improve cardiorespiratory fitness and is expected to help in reducing adiposity [14]. In general, overweight and obese children are likely to have relatively low physical fitness levels. A previous study has shown the negative association between BMI and physical fitness [15]. A study among 7–11 years old Brazilian children found that overweight and obese children perform poorly in cardiorespiratory fitness, and muscular strength and endurance [16]. Furthermore, previous studies have shown that moderate to higher levels of cardiorespiratory fitness are associated with lower abdominal adiposity [17], whereas body fat percentages and BMI have negative associations with physical fitness [18]. Another study showed that cardiorespiratory fitness was significantly higher in normal weight children aged 8–13 years and has a negative association with BMI [19]. A physically fit individual would be able to carry out daily tasks with more energy and less fatigue, hence can spend more time in physical activity [20]. Thus, increase in physical fitness is likely to benefit overweight and obese children. Therefore, the aim of this study is to investigate the effects of MyBFF@school program on the cardiorespiratory fitness of overweight and obese primary schoolchildren.

## Methods

### Study design

**Fig. 1:**
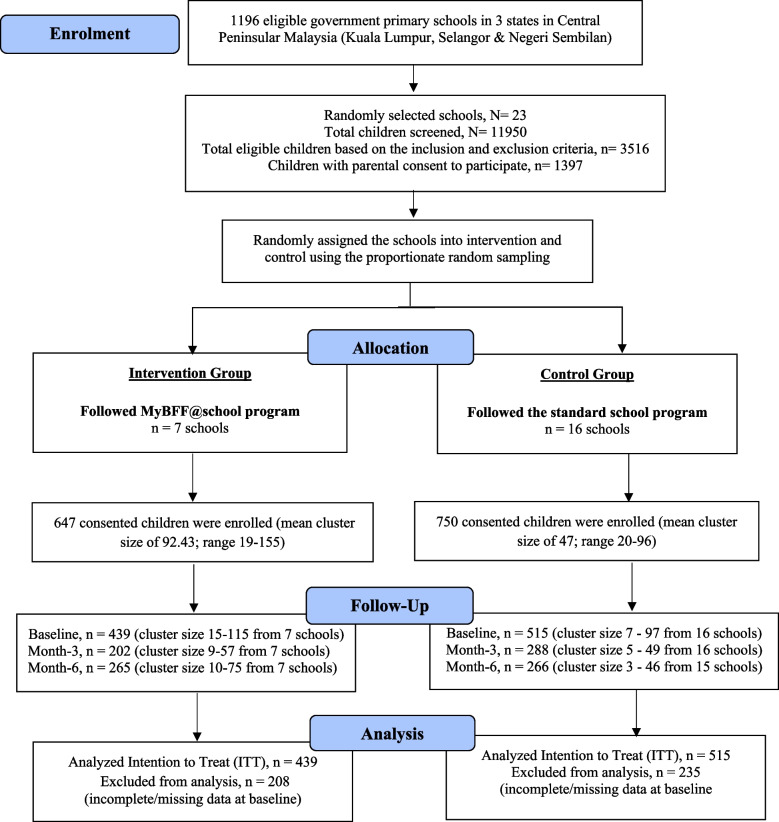
CONSORT diagram for small-sided games component in MyBFF@school

We conducted a randomized cluster-controlled trial on schoolchildren as described in Mokhtar et al. [13]. A total of 23 government primary schools were randomly selected across three states in central Peninsular Malaysia (Wilayah Persekutuan Kuala Lumpur, Selangor, and Negeri Sembilan). These schools were randomized into the MyBFF@school intervention (seven schools) and control groups (16 schools) (Fig. 1). Schoolchildren aged 9–11 years (Standard Three to Five) with a body mass index (BMI) *z*-score greater than + 1 SD (WHO) were eligible for the study. The MyBFF@school intervention group participated in a six months intervention program composed of sessions of 30 min of small-sided games (SSG) replacing regular physical education sessions, and an additional 1 h of nutrition alternating with psychology education replacing their weekly co-curriculum activities. The standard co-curriculum activities [21] were conducted once a week, usually on Wednesday afternoon i.e. during school hours. Hence, a typical week in the intervention group would have two sessions of SSG and one session of either nutrition or psychology module. The control group underwent the standard physical education sessions and co-curriculum activities. All sessions were done within school hours. The eligibility, assessment and module of the participants were described in detail in Mokhtar et al. [13]. The calendar year in 2016 started with school opening on January 4th, 2016. The first two weeks of school were hectic with administrative matters for the school, schoolchildren and their parents. We started collecting baseline data at the end of January until mid-February 2016. The intervention started in mid-February and ended in mid-August. Within this period, there were holidays: mid-term break in March for one week; mid-term break at the end of May and early June for two weeks. The month-3 data were collected in mid-May before their mid-term break and the month-six data were collected between mid-August until early September 2016.

### MyBFF@school physical activity component

The physical activity component of MyBFF@school program is the small-sided games (SSG). The SSG sessions were conducted during the regular physical education periods. Each session of SSG lasted for 30 min and performed twice per week giving a total of 60 min per week. During the SSG session, the schoolchildren were divided into several teams with each team consisting of about four to seven players. Each team has schoolchildren from different classes and gender. Each SSG session started with 5 min of warm up (mainly dynamic stretching and running), followed by basic skills (kicking, dribbling, passing, shooting) and gameplay (football, handball, and fun games) for 20 min. The last 5 min were spent on cooling down. The SSG were played in a smaller field measuring approximately 14 m × 9 m. The main sport played was football besides handball and other fun games (e.g. monkey ball, dodgeball). A trained personnel (research assistant) facilitated the SSG throughout. The trained personnel had undergone a few training sessions prior to the study period, and trained by a qualified football coach who was also a school teacher. First aid kit for emergencies was available at the sideline during each SSG session. Other safety measures such as checking on self-reported health status, weather and equipment assessment, proper sports attire, and hydration were strictly adhered for SSG. In the event of bad weather or haze, SSG was played indoors or in a roofed area e.g. an open hall. In addition, medical doctors from the research team were available to render help by referral in case of injury or illness.

SSG differed from the standard physical education program as the emphasis was more on play, touch and run within the small field. In a previous study, SSG played for 40 min, three times a week (a total of 120 min/week), showed improvement in cardiovascular fitness, muscle growth and strength that were able to be maintained for a long period of time in children [22]. In our study, the SSG was played half of this volume as the allowed time by school authority was the time slot for Physical Education classes. Meanwhile, the standard program focused more on developing basic movement and game skills and emphasized on correct technique. From our observation, there was less game play in the standard school program as compared to SSG. The sports in the syllabus included several popular games including football, netball, racquet sports, and track and field [23, 24]. We hypothesized that the standard program may not be sufficient for overweight and obese children as greater amount of physical activity is required for weight loss and maintaining it.

### Anthropometric measurements

Height was measured using a mobile stadiometer (Seca 217, Seca GmbH & Co. KG, Germany) and weight was measured using a digital weight scale (Tanita HD-319, Tanita Co., Japan). Body mass index corrected for age, or BMI *z*-score, was calculated from the height and weight values using the WHO anthropometric calculator (World Health Organization). The weight categories namely overweight, obese, and morbidly obese were defined by BMI *z*-scores above 1, 2, and 3 SDs, respectively, adjusted for age and gender according to the WHO BMI chart (2008) [25]. Abdominal obesity was defined as a WC of the 90th percentile or higher on the Malaysian WC chart [26]. From the total screened, 3516 of them had BMI *z*-score more than + 1 SD (WHO standard) and eligible for the study. Of these, 1397 assented and given consent by parent/guardian to participate. Their body composition including percentage body fat and skeletal muscle mass were then measured using bioelectrical impedance analyzer (InBody 720, InBody Co. Ltd., Seoul, Korea).

### Physical activity, physical fitness and cardiorespiratory fitness

Physical activity is defined by the World Health Organization (WHO) as any bodily movement produced by skeletal muscles that requires energy expenditure [27] and is commonly described by four dimensions i.e. frequency, duration, intensity, and mode [28]. Whereas physical fitness comprises cardiorespiratory endurance, muscle endurance and muscle strength, flexibility, balance, agility, and coordination [29]. Physical activity is known to improve physical fitness particularly cardiorespiratory and muscular fitness [27]. Cardiorespiratory fitness is sometimes referred as cardiorespiratory endurance, cardiovascular fitness, aerobic capacity, and aerobic fitness [30]. Thus, it can be derived that physical activity, particularly moderate and vigorous intensity, could improve cardiorespiratory fitness which is a component of physical fitness. For the purpose of our study, cardiorespiratory fitness was measured as a physical fitness score by using modified Harvard step test.

### Modified Harvard step test

The Harvard step test is an aerobic fitness test developed during World War II in the Harvard Fatigue Laboratories which is easy to perform and requires minimal equipment [31]. Meanwhile, a modified Harvard step test has been modified from the original Harvard step test which commonly used to test dynamic fitness [32] i.e. the physical potential before sports training, and monitoring physical fitness.

The modified Harvard step test has been regularly used to measure the cardiorespiratory fitness in the general population including schoolchildren and adolescents [33–35]. The test has been shown to be moderately reliable with intraclass correlation coefficient of 0.62 and is recommended from other aerobic (cardiorespiratory) fitness tests to be used in sports and occupational settings. The other more reliable tests like 1-mile track walk test, 12-minute run test and interval shuttle runs require a larger area, time consuming and influenced by the subject’s motivation [35]. The cardiorespiratory fitness is calculated based on heart rate response toward a standard, submaximal exercise [36]. Furthermore, the post-exercise heart rate has been shown to be useful in determining cardiorespiratory fitness in children aged 6–12 years old [37]. Statistically significant correlations were observed between VO_2_max and the step test (*r* = −0.549) in children aged 10–17 (mean age (SD) was 12.8 (1.9) years) [38]. Various submaximal step tests have been validated for the use in children and adolescents in the literature [39–41]. In the modified Harvard step test, the sum of three post-exercise pulse counts are used. Participants would undergo three stages of the test: resting, stepping and post-exercise rest. During the resting stage, the participant sat on a chair for 5 min and a finger pulse oximeter (Nonin GO2 9570, Nonin Medical Inc., USA) was applied on the participant’s finger to monitor their pulse rate. Next, in the stepping stage, the participant was instructed to step up and down with both feet over a step box with 30 cm height and 42 cm width [42, 43]. The tempo followed a 120 beats per minute metronome guiding the participant to perform 30 steps per min for 5 min or until the participant is unable to continue. The pulse rate and oxygen saturation were monitored and recorded throughout the test. The test would be stopped if the participant’s heart rate was above 200 beats per minute, had difficulty in breathing, SpO_2_ less than 90% or unable to finish. The test was conducted by trained personnel led by sports medicine doctors. Upon completion, the participant was instructed to sit down and rest. Finally, in this third (post-exercise) stage, the heart rate and oxygen saturation were taken at 0, 1, and 2 min. Afterwards, the physical fitness score (PFS) was calculated using the following formula: (total duration in seconds divided by the sum of post-exercise heart rate at 0, 1, and 2 min) × 100 [33, 43–45]. For example, a participant who completed 5 min of the test with post-exercise heart rates of 140, 130, 120 at 0, 1 and 2 min bpm respectively scores a PFS [(5 × 60 s) / (140 + 130 + 120)] × 100 = 76.92. For the purpose of this study, the score was rounded at two decimal places for the use of the analysis.

### Statistical analysis

All outcomes were analyzed according to the intention-to-treat principle and were reported after three and six months from baseline. Analysis were based on intention-to-treat, and multiple imputation methods were applied for the missing data at month-three and month-six. All statistical analyses were carried out using IBM Statistical Package for Social Science for Windows, Version 24.0. Normality of continuous data was tested using the Kolmogorov–Smirnov test. All continuous variables were expressed as mean ± standard deviation (SD). Baseline means were compared between groups using the independent samples *t*-test. Mixed linear models with group (intervention/control) as fixed effect and school as random effect were used to evaluate the effectiveness after three and six months of intervention. The intracluster correlation coefficient (ICC) was also estimated.

## Results

The total number of schoolchildren who assented and were given consent by their parents or guardian was 1397. Of these 1397 children only 954 children completed the baseline physical assessment with 439 children were in the intervention group, while 515 in the control group. The remaining children did not complete the study due to being absent from school, self-reported unwell, time constraint and not keen for physical fitness testing (voluntary refusal) on the assessment day. The baseline characteristics of the groups are presented in Table 1. All baseline characteristics of the intervention and control groups did not differ significantly except for the age in which the intervention is slightly less than the control (9.82(0.85) years vs. 9.93(0.85), *p* = 0.04). Tables 2, 3 and 4 present the mean heart rate (HR) at 0-min, and the HR recovery (HRR) at min-1 and min-2, at three timelines i.e. at baseline, month-3 and month-6. These tables give an overall picture of the data collected.

**Table 1:** Baseline characteristics of the participants (SD) (*N* = 954)

	Intervention (*n* = 439)	Control (*n* = 515)	*p*-value
**Number of schools**	7	16	
**Number of participants per school**,** mean (SD)**	45.09 (1.63)	67.41 (4.29)	
**Number of participants per school**,** median (range)**	45 (15–115)	68 (7–97)	
**Mean age**^*a*^, **years (SD)**	9.82 (0.85)	9.93 (0.85)	0.04
**Height (cm)**^*a*^	138.69 (7.71)	138.84 (7.66)	0.89
**Weight (kg)**^*a*^	45.01 (10.02)	45.51 (10.35)	0.89
**BMI (kg/m2)**^*a*^	23.18 (3.45)	23.42 (3.64)	0.51
**BMI** ***z*** **-score (SD)**^*a*^	2.23 (0.80)	2.23 (0.82)	0.76
**Percentage body fat (%)**^*a*^	36.71 (6.50)	37.50 (6.50)	0.99
**Skeletal muscle mass (kg)**^*a*^	14.57 (2.85)	14.59 (2.87)	0.81
**Overall physical fitness score**^*a*^	69.5 (6.3)	69.6 (6.6)	0.74
**Gender**,** n (%)**^***b***^			0.45
* Boys*	234 (53.3)	287 (55.7)	
* Girls*	205 (46.7)	228 (44.3)	
**Weight categories**,** n (%)**^***b***^			0.68
* Overweight*	193 (44.0)	213 (41.4)	
* Obese*	176 (40.1)	220 (42.7)	
* Morbidly obese*	70 (15.9)	82 (15.9)	

^*a*^Independent *t*-test, ^*b*^chi-squared test

**Table 2:** The mean heart rate (HR) and heart rate recovery (HRR) at baseline

	Intervention				Control			
	*N*	Mean (SD) HR at 0-min (bpm)	Mean (SD) HRR at 1-min^a^ (bpm)	Mean (SD) HRR at 2-min^b^ (bpm)	*N*	Mean (SD) HR at 0-min (bpm)	Mean (SD) HRR at 1-min^a^ (bpm)	Mean (SD) HRR at 2-min^b^ (bpm)
**Overall**	439	172 (15)	33 (16)	48 (17)	515	168 (15)	27 (16)	44 (17)
**Gender**								
Boys	234	169 (14)	35 (17)	49 (16)	287	166 (15)	29 (17)	45 (16)
Girls	205	174 (15)	30 (15)	46 (17)	228	171 (15)	25 (15)	43 (17)
**Weight status**								
Overweight	193	170 (15)	33 (17)	48 (18)	213	167 (15)	30 (16)	46 (15)
Obese	176	173 (15)	33 (15)	49 (15)	220	169 (16)	27 (17)	44 (18)
Morbidly Obese	70	174 (13)	30 (16)	46 (16)	82	168 (16)	23 (12)	41 (15)
**Location**								
Urban	209	170 (16)	32 (17)	47 (18)	331	169 (15)	27 (15)	44 (17)
Rural	230	173 (14)	33 (15)	48 (15)	184	168 (15)	28 (18)	44 (16)

bpm beats per min, HR Heart rate, HRR Heart rate recovery, SD Standard deviation

^*a*^Mean HRR at 1-min = Mean (SD) HR at 0-min - Mean (SD) HR at 1-min

^*b*^Mean HRR at 2-min = Mean (SD) HR at 0-min - Mean (SD) HR at 2-min

**Table 3:** The mean heart rate (HR) and heart rate recovery (HRR) at month-3

	Intervention				Control			
	*N*	Mean (SD) HR at 0-min (bpm)	Mean (SD) HRR at 1-min^a^ (bpm)	Mean (SD) HRR at 2-min^b^ (bpm)	*N*	Mean (SD) HR at 0-min (bpm)	Mean (SD) HRR at 1-min^a^ (bpm)	Mean (SD) HRR at 2-min^b^(bpm)
**Overall**	439	163 (18)	29 (16)	45 (16)	515	164 (16)	29 (17)	44 (16)
**Gender**								
Boys	234	160 (18)	29 (16)	45 (16)	287	163 (15)	30 (17)	46 (16)
Girls	205	168 (17)	28 (16)	45 (17)	228	166 (17)	27 (15)	42 (16)
**Weight status**								
Overweight	193	164 (19)	30 (16)	45 (17)	213	162 (17)	30 (17)	44 (17)
Obese	176	162 (18)	27 (18)	43 (16)	220	166 (16)	28 (16)	44 (16)
Morbidly Obese	70	165 (13)	27 (13)	45 (15)	82	168 (15)	27 (15)	44 (14)
**Location**								
Urban	209	163 (19)	27 (17)	44 (17)	331	164 (17)	28 (16)	44 (16)
Rural	230	164 (17)	30 (15)	46 (16)	184	165 (15)	31 (17)	45 (16)

*bpm* beats per min, *HR* Heart rate, *HRR* Heart rate recovery, *SD* Standard deviation

^*a*^Mean HRR at 1-min = Mean (SD) HR at 0-min - Mean (SD) HR at 1-min

^*b*^Mean HRR at 2-min = Mean (SD) HR at 0-min - Mean (SD) HR at 2-min

**Table 4:** The mean heart rate (HR) and heart rate recovery (HRR) at month-6

	Intervention				Control			
	*N*	Mean (SD) HR at 0-min (bpm)	Mean (SD) HRR at 1-min^a^ (bpm)	Mean (SD) HRR at 2-min^b^ (bpm)	*N*	Mean (SD) HR at 0-min (bpm)	Mean (SD) HRR at 1-min^a^ (bpm)	Mean (SD) HRR at 2-min^b^ (bpm)
**Overall**	439	160 (17)	29 (16)	44 (16)	515	162 (17)	28 (16)	45 (16)
**Gender**								
Boys	234	159 (18)	28 (15)	44 (16)	287	159 (17)	29 (16)	45 (17)
Girls	205	162 (15)	29 (17)	44(16)	228	166 (16)	28 (15)	45 (16)
**Weight status**								
Overweight	193	158 (18)	30 (18.82)	44 (16)	213	162 (16)	31 (18)	48 (17)
Obese	176	161 (16)	28 (14)	45 (16)	220	161 (17)	26 (14)	44 (16)
Morbidly Obese	70	162 (16)	27 (16)	43 (16)	82	164 (17)	28 (13)	43 (15)
**Location**								
Urban	209	159 (18)	30 (17)	45 (15)	331	163 (15)	28 (15)	45 (16)
Rural	230	161 (16)	28 (16)	43 (17)	184	161 (18)	28 (16)	45 (18)

*bpm* beats per min, *HR* Heart rate, *HRR* Heart rate recovery, *SD* Standard deviation

^*a*^Mean HRR at 1-min = Mean (SD) HR at 0-min - Mean (SD) HR at 1-min

^*b*^Mean HRR at 2-min = Mean (SD) HR at 0-min - Mean (SD) HR at 2-min

## Effectiveness of intervention after three months

There was overall within-group improvement of PFS in both groups. The control has slightly better results and more significant changes. However, when compared between-groups, the mean difference of PFS was not statistically significant: (−0.15, 95%CI −0.75, 0.45, *p* = 0.83).

In boys neither within- nor between-group showed any significant improvement of PFS in both groups. Whereas, the girls showed significant within-group improvement in the intervention and control. Nevertheless, when compared between-groups, the girls did not yield a significant difference (−0.27, 95%CI −1.27, 0.73, *p* = 0.81).

In the weight categories, only obese schoolchildren from the control group showed within-group significant improvement (1.80, 95%CI 0.43, 3.17). Nevertheless, the effect was diluted when compared between-groups (−0.05, 95%CI −1.03, 0.92), *p* = 0.93). There was no significant improvement observed for other weight categories.

Location-wise, there were mixed results. The urban schoolchildren in the control group showed significant within-group changes of PFS (1.40, 95%CI 0.17, 2.64). In contrast, the rural schoolchildren in the intervention group showed within-group improvement (1.68, 95%CI 0.21, 3.14). However, the urban and rural locations did not show significant between-group differences (Table 5).

**Table 5:** Differences for physical fitness score between control and intervention groups at baseline and at month-3

	Intervention				Control						
	*N*	Mean (SD) baseline	Mean (SD) month-3	Change within-group (Month-3, baseline) Mean difference (95% CI)	*N*	Mean (SD) baseline	Mean (SD) month-3	Change within-group (Month-3, baseline) Mean difference (95% CI)	Change between-group (Intervention, control) Mean difference (95% CI)	*p*-value	ICC
**Overall**	439	69.46 (6.29)	70.37 (6.86)	1.14 (0.09, 2.20)	515	69.59 (6.55)	70.51 (6.36)	1.29 (0.26, 2.33)	−0.15 (−0.75, 0.45)	0.83	0.001
**Gender**											
* Boys*	234	71.14 (6.24)	72.15 (7.03)	0.64 (−0.75, 2.04)	287	71.09 (6.47)	71.75 (6.74)	0.63 (−0.62, 1.89)	−0.07 (−0.98, 0.83)	0.83	0.008
* Girls*	205	67.53 (5.80)	69.35 (5.69)	1.72 (0 0.42, 3.03)	228	67.71 (6.16)	69.84 (6.66)	2.13 (0.74, 3.52)	−0.27 (−1.27, 0.73)	0.81	0.013
**Weight category**											
* Overweight*	193	70.48 (6.67)	70.75 (6.81)	0.53 ( −0.88, 1.95)	213	70.74 (6.66)	72.29 (6.71)	1.02 (−0.52, 2.58)	−0.16 (−1.28, 0.94)	0.97	0.004
* Obese*	176	69.07 (6.23)	70.54 (6.91)	1.57 (−0.24, 3.39)	220	68.88 (6.32)	70.74 (6.56)	1.80 (0.43, 3.17)	−0.05 (−1.03, 0.92)	0.93	0.000
* Morbidly Obese*	70	67.60 (4.74)	70.00 (7.47)	1.67 (−1.44, 4.80)	82	68.52 (6.51)	69.31 (6.12)	0.87 (−1.61, 3.36)	−0.68 (−2.43, 1.05)	0.26	0.006
**Location**											
* Urban*	209	69.97 (6.21)	70.57 (6.89)	0.56 (−1.10, 2.22)	331	69.43 (6.51)	70.97 (6.71)	1.40 (0.17, 2.64)	0.07 (−0.79, 0.94)	0.45	0.002
* Rural*	230	68.99 (6.35)	70.61 (6.72)	1.68 (0.21, 3.14)	184	69.89 (6.63)	71.13 (6.73)	1.10 (−0.37, 2.58)	−0.35 (−1.34, 0.62)	0.30	0.000

*CI* Confidence interval, *ICC* Intraclass coefficient, *SD* Standard deviation

### Effectiveness of intervention after six months

In overall, there was significant within-group improvement for both intervention and control (Table 6). In the intervention group, the MD of PFS was found to be slightly higher (2.71, 95%CI 1.65, 3.77) than the control (2.10, 95%CI 1.10, 3.11). However, when comparing between-groups, the effect was too small and not statistically significant (0.05, 95%CI −0.98, 1.07, *p* = 0.69).

**Table 6:** Differences for physical fitness score between control and intervention groups at baseline and at month-6

	Intervention				Control						
	*N*	Mean (SD) baseline	Mean (SD) month-6	Change within group (Month-6, baseline) Mean difference (95% CI)	*N*	Mean (SD) baseline	Mean (SD) month-6	Change within group (Month-6, baseline) Mean difference (95% CI)	Change between group (Intervention, control) Mean difference (95% CI)	*p*-value	ICC
**Overall**	439	69.46 (6.29)	72.32 (6.92)	2.71 (1.65, 3.77)	515	69.59 (6.55)	71.79 (7.25)	2.10 (1.10, 3.11)	0.05 (−0.98, 1.07)	0.69	0.044
**Gender**											
* Boys*	234	71.14 (6.24)	72.99 (7.49)	1.84 ( 0.51, 3.16)	287	71.09 (6.47)	72.81 (7.41)	1.39 (−0.02, 2.80)	0.06 (−1.35, 1.46)	0.86	0.015
* Girls*	205	67.53 (5.80)	71.54 (6.15)	3.71 (2.29, 5.14)	228	67.71 (6.16)	70.50 (6.86)	3.01 (1.68, 4.34)	0.10 (−1.31, 1.51)	0.74	0.019
**Weight category**											
* Overweight*	193	70.48 (6.67)	72.18 (6.64)	1.99 (0.32, 3.66)	213	70.74 (6.66)	72.20 (7.25)	1.91 (0.24, 3.58)	0.15 (−1.07, 1.36)	0.93	0.006
* Obese*	176	69.07 (6.23)	72.79 (7.03)	3.48 (1.91, 5.05)	220	68.88 (6.32)	71.78 (7.46)	3.33 (1.80, 4.86)	0.28 (−0.98, 1.55)	0.75	0.013
* Morbidly Obese*	70	67.60 (4.74)	70.46 (6.47)	2.43 (−0.25, 5.11)	82	68.52 (6.50)	70.34 (7.81)	2.24 (0.01, 4.47)	−0.79 (−2.74, 1.15)	0.47	0.036
**Location**											
* Urban*	209	69.97 (6.21)	72.25 (7.37)	2.49 (1.07, 3.91)	331	69.43 (6.51)	71.78 (7.13)	2.08 (0.87, 3.30)	0.61 (−0.56, 1.77)	0.47	0.005
* Rural*	230	68.99 (6.35)	72.38 (6.51)	2.92 (1.29, 4.54)	184	69.89 (6.63)	71.80 (7.48)	2.14 (0 0.61, 3.68)	−0.69 (−2.52, 1.14)	0.17	0.001

*CI* Confidence interval, *ICC* Intraclass coefficient, *SD* Standard deviation

In boys, there was a significant improvement of PFS in the intervention group (1.84, 95%CI 0.51, 3.16), but not in the control (1.39, 95%CI −0.02, 2.80). Whereas in girls, both intervention and control showed significant within-group improvement of PFS (3.71_intervention_, 95%CI, 2.29, 5.14); 3.01_control_, 95%CI 1.68, 4.34) respectively. Nevertheless, we did find any significant improvement of PFS for both boys and girls when compared between intervention and control groups.

For the weight categories, within-group improvements of PFS were observed in all categories for both intervention and control. However, these effects did not remain significant when comparing between intervention and control groups.

For school location, the urban schoolchildren in both intervention and control groups showed significant within-group improvement (2.49_intervention_, 95%CI 1.07, 3.91 vs. 2.08_control_, 95%CI 0.87, 3.30), respectively. However, these effects were diluted when comparing between groups. Similar patterns were observed among rural schoolchildren. We found a significant within-group improvement of PFS in intervention and control (2.92_intervention_, 95%CI 1.29, 4.54 and 2.14_control_, 95%CI 0.61, 3.68). Comparing intervention and control, the mean difference was small and remained not significant (−0.69, 95%CI −2.52, 1.14, *p* = 0.17).

## Discussion

This study found that the program did not result in significant cardiorespiratory fitness, but projected a positive pattern for the intervention group after six months. In the first three months, there was significant improvement in cardiovascular physical fitness within the group in almost all parameters for both the intervention and control groups. Namely, there were significant within-group improvements in overall, gender and weight categories. By six months, the within group changes maintain almost the same pattern, but the mean differences favor the intervention. Nevertheless, as per month-3, these mean differences remained not significant. Hence, both MyBFF@school and the control groups showed significant within group improvement in cardiorespiratory physical fitness over three and six months. We did not record the activities in Physical Education classes in control schools that could have explained the similar improvement seen in the control group. In addition, the after school physical activities were not recorded which could also contribute to the improvement in both groups. We did not measure the actual load of SSG and the standard physical education. It is possible that the children did not reach moderate-to-vigorous intensity during SSG. The actual measurement on volume by accelerometer and intensity by heart rate measurement could have explained whether the SSG and the standard Physical Education had reached the desired volume and intensity [46, 47]. This could also explain the compliance. We were limited by resources, namely financial and manpower, to do this. The load was also confounded by the fact that the permitted time for SSG and control were just 60 min per week. Having said this, the pattern of changes indicates that long term intervention i.e. six months or more of MyBFF@school has the potential to yield better results in cardiorespiratory physical fitness score compared to the standard program. This would help in increasing physical activity in tandem with WHO guidelines on physical activity and sedentary behavior (2020) [48]. A recent study showed that moderate or vigorous intensity of physical activity is positively correlated with cardiorespiratory fitness in children aged 7 to 12 year old. The authors suggested this was mediated by reducing adiposity [49]. Poitras et al. (2016) did a systematic review on the relationship between physical activity and health indicators in school-aged children and youth. They analysed 33 studies (of 162 studies reviewed) and found that all intensity of physical activity was favorably associated with physical fitness particularly cardiorespiratory and muscular fitness in school-aged children and youth with more consistent and robust for higher intensity physical activity [50]. Another study on 753 children aged 10–14 years found, among others, better cardiorespiratory fitness was associated with higher levels of physical activity [51]. This is beneficial in the long run as higher level of physical fitness in obese children is shown to have healthier cardiovascular profile compared to overweight and normal weight peers with low fitness [52]. Increased aerobic fitness level also may protect against worsening of childhood obesity as it was shown to attenuate metabolic syndrome score and could reduce the risk of obesity-related comorbidities [53]. Furthermore, previous study has shown an inverse association between physical fitness and lower abdominal obesity as measured by waist circumference [54].

One of the reasons for it to be potentially successful is because SSG has been shown to be highly enjoyable [55] and this could be the reason for adherence and high engagement. Furthermore, team sports as promoted in SSG has been shown to increase enjoyment in children aged 8–10 years compared with individual sport participation [56]. It dictated the children to move around at high intensity while playing games continuously for 30–40 min per session. Fewer players and smaller fields in SSG increased the children’s participation and enjoyment as more ball possession, kicking or throwing, dribbling and dodging. Furthermore, in this SSG, we added multiple games besides football i.e. handball and fun games which would have contributed to the increase in physical activity and fitness [57]. A previous study revealed that boys who spent many hours per week in multiple sports were significantly fitter than boys who spent a few hours per week in a single sport [58]. One of the findings by The Malaysian National Health and Morbidity Survey (NHMS) 2012 was that, only 8.9% of children aged 10 to 17 years were fully engaged in physical education classes [59]. If the engagement is increased by MyBFF@school program, this would likely to improve the physical activity and, over time, the cardiorespiratory fitness.

The mean differences between intervention and control were not significant for boys and girls in both month-three and -six. It is possible that puberty could have influenced the outcome of this study. Puberty in boys affects aerobic fitness positively. This is contributed by alteration in body composition during puberty and improvement in maximal tissue oxygen consumption kg/min or better known as VO_2_max. The body composition in pubertal boys presents a significant rise in growth of bone, stature and muscle mass and at the same time loss of fat in limbs under the influence of testosterone. The VO_2_max improves due to increase in maximal stroke volume, increase in lung size, skeletal muscle mass, and muscle capillarization [60]. Nevertheless, we do not want to be over-speculative on puberty influence in boys in our study as we did not control puberty in the analysis.

In girls, puberty, which dictates sexual maturation, tends to affect differently. A study on 3201 boys and girls, aged 8–14 years, found that early sexual maturation was positively associated with overweight and obesity in girls but was reversed in boys [61]. A more recent study found that pubertal changes in obese girls may occur earlier than normal weight girls [62]. On top of that, biological maturation was found to moderate the relationship between cardiorespiratory fitness in boys but not girls. Pre-puberty cardiorespiratory fitness presents no gender difference, but the impact of puberty on physical fitness among girls is lower than in boys [63]. Girls tend to increase in percentage body fat during puberty [64], hence limiting the gains in cardiorespiratory fitness. In this study, we found that the cardiovascular fitness in girls improved in both groups significantly. The improvement was against the hypothesis that the cardiovascular fitness gain in girls would not be as much as the boys during and in early puberty. This finding is encouraging and supports both programs (MyBFF@school and standard school). At the same time, our findings agree with previous meta-analysis that concluded physical activity interventions in girls aged 5–11 years resulted in small positive albeit significant effects [65]. Nevertheless, we could not show the advantage of MyBFF@school over the standard program for girls.

We compared the changes of cardiovascular physical fitness between MyBFF@school urban schoolchildren and the control; and similarly on the rural schoolchildren. We did not find any significant differences although there was a slight favorable mean difference to the intervention group at month-3 and month-6 in urban schoolchildren. This could be potentially beneficial as Malaysian NHMS 2019 has shown that urban children have a higher prevalence of overweight and obesity [6]. A Malaysian local study via a self-reported questionnaire on 3,798 schoolchildren aged 12 years, found several socioeconomic factors to be predictors of obesity in the urban schoolchildren, for example, family size and household income [66]. Meanwhile, in the rural schoolchildren of our study, both groups showed within-group improvement although the mean differences were slightly favorable to the control. There is insufficient information to explain the reason for this especially since it is related to the local setting. A local cross-sectional study found that 33.4% of the variation in BMI of schoolchildren (*n* = 400 schoolchildren aged 9 to 11 years) was explained by certain factors including, among others, health professional involvement, simple exercise before class, availability of policy on physical activity and training teacher as a role model [67]. Another local study in Terengganu, a state in Malaysia, highlighted that rural school scored better in terms of physical environment to support physical activity e.g. in health, nutrition and physical activity programs and school facilities [68]. Unfortunately, our study did not collect information on all these factors to discuss in detail the different outcomes seen based on school locations.

Detraining effects may occur due to prolonged cessation of exercises e.g. school holidays. The six months intervention in this study included school holidays i.e. a total of three weeks within the period of study. The holidays were mid-term break in March (March 12th until March 20th ) for one week; and mid-term break at the end of May and early June for two weeks (May 28th, until June 13th, 2016). In our study, the potential effect of detraining may be diluted as the school breaks were relatively short. In a systematic review on the effect of detraining of obese children (*n* = 330, age 6–12 years old) who underwent between 12 and 48 weeks of physical exercise programs followed by between 12 and 48 weeks of detraining, concluded that detraining did not lead to significant loss of the gains in the lipid profile particularly high density lipoprotein (HDL). The authors concluded that the period of training of 36 to 48 weeks were required for a protection against detraining and highlighted the heterogeneity of the detraining period which would require a consensus [69]. Another study looked at detraining effects on adolescent boys (age 13 ± 1.04 years). The study found no loss on the aerobic fitness gain (measured by VO_2_max) for the eight weeks exercising groups (either resistance only or combined resistance and endurance) against the control (no exercise program) who underwent detraining of 12 weeks [70]. In one review article, the training-induced gains may occur after two to four weeks detraining in concurrent resistant and aerobic training among young adults aged between 18 and 35 years [71]. Research of detraining effects especially related to cardiorespiratory fitness in children is lacking and we could not derive any conclusion for this population. In our study, the potential effect of detraining may possibly confound the outcome of the intervention although the extent is not clear as the school breaks were relatively short.

### Limitations

The period of intervention may be relatively short to show more positive results. Several intervention studies need at least one year since a shorter period of intervention may not have significant effects on overweight and obese children [72]. Even in normal children and adolescents, school-based physical activity intervention takes a long time to yield a positive outcome [73]. It could be the same or worse for overweight and obese children. A meta-analysis assessing effectiveness of interventions in aerobic fitness adjusted for weight in obese children found programs based on aerobic exercise had a moderate positive effect on physical fitness and lasting more than 12 weeks (3000 min per session) in three sessions per week (more than 60 min per session) obtained better result [74]. Another meta-analysis that analyzed the duration of implementation and found that intervention that applied more than 1–2 years or longer than two years yielded better than programs less than six months [75]. In tandem, weight loss in obesity intervention programs requires a long duration as highlighted by a Cochrane review that revealed low quality evidence of small and short term reduction for children aged 6 to 11 years [76]. This is further supported by current guidelines of obesity intervention emphasising longer intervention yielded better results [77].

In addition, the time allocated by the schools for the SSG was relatively short: only twice a week and for 30 min per session. This was required to conform to the school curriculum, but may have affected the benefit of physical activity in SSG. Indeed, this falls short of the guideline for physical activity in children recommended by the American College of Sports Medicine, which suggests that students should engage in 60 min of moderate to vigorous activity per day [78]. Unfortunately, other forms of physical activity done by the schoolchildren out of their intervention or standard program for example after school activities were not accounted for. Furthermore, the intervention program could be better if attention was given to progression of intensity as highlighted in a systematic review article [79]. We did not increase the intensity as it required more monitoring for example by heart rate measurement of the sessions and adjustment by increasing the tempo of the gameplay, or increasing the duration and frequency of the SSG. Future studies should include the after-school activity record, and consider intensity monitoring and progression as these could contribute to the outcome of the study.

We did not capture family support in this study. Some studies have advocated that a multi-disciplinary approach including family participation is crucial for the effectiveness of an obesity intervention program. Parents are imperative in the development of children’s behavior and could have contributed to better outcomes [80]. Furthermore, engaging support from friends and caring adults were shown to contribute to a desirable result [81]. However, recruiting and engaging parents in such interventions can be a considerable challenge for researchers and practitioners [82]. Future studies should look into this and incorporate family support and friends in the school-based intervention.

## Conclusion

MyBFF@school program showed positive trend in cardiorespiratory fitness changes especially after six months. MyBFF@school intervention program has the potential to combat obesity in primary schoolchildren and should be at least six months.

## Data Availability

All relevant data are within the paper.

## Abbreviations

ANCOVA: Analysis of covariance
ANOVA: Analysis of variance
BMI: Body mass index
CI: Confidence interval
ICC: Intracluster correlation coefficient
MD: Mean difference
NHMS: National Health and Morbidity Survey
PFS: Physical fitness score
SD: Standard deviation
SSG: Small-sided games
WHO: World Health Organization
